# Cardioprotective effects of genetically engineered cardiac stem cells by spheroid formation on ischemic cardiomyocytes

**DOI:** 10.1186/s10020-019-0128-8

**Published:** 2020-01-31

**Authors:** Han Saem Jeong, Chi-Yeon Park, Jong-Ho Kim, Hyung Joon Joo, Seung-Cheol Choi, Ji-Hyun Choi, I-Rang Lim, Jae Hyoung Park, Soon Jun Hong, Do-Sun Lim

**Affiliations:** 1 Department of Cardiology, Cardiovascular Center, Korea University College of Medicine, Seoul, Republic of Korea; 2 Department of Cardiology, Cardiovascular Center, Korea University College of Medicine, Seoul, Republic of Korea

**Keywords:** Sca-1+ cardiac stem cell, Cardiac sphere, acute myocardial infarction, Cardioprotection, CXCR4, SDF-1α

## Abstract

**Background:**

Sca-1+ cardiac stem cells and their limited proliferative potential were major limiting factors for use in various studies.

**Methods:**

Therefore, the effects of sphere genetically engineered cardiac stem cells (S-GECS) inserted with telomerase reverse transcriptase (TERT) were investigated to examine cardiomyocyte survival under hypoxic conditions. GECS was obtained from hTERT-immortalized Sca-1+ cardiac stem cell (CSC) lines, and S-GECS were generated using poly-HEMA.

**Results:**

The optimal conditions for S-GECS was determined to be 1052 GECS cells/mm^2^ and a 48 h culture period to produce spheroids. Compared to adherent-GECS (A-GECS) and S-GECS showed significantly higher mRNA expression of SDF-1α and CXCR4. S-GECS conditioned medium (CM) significantly reduced the proportion of early and late apoptotic cardiomyoblasts during CoCl_2_-induced hypoxic injury; however, gene silencing via CXCR4 siRNA deteriorated the protective effects of S-GECS against hypoxic injury. As downstream pathways of SDF-1α/CXCR4, the Erk and Akt signaling pathways were stimulated in the presence of S-GECS CM. S-GECS transplantation into a rat acute myocardial infarction model improved cardiac function and reduced the fibrotic area. These cardioprotective effects were confirmed to be related with the SDF-1α/CXCR4 pathway.

**Conclusions:**

Our findings suggest that paracrine factors secreted from transplanted cells may protect host cardiomyoblasts in the infarcted myocardium, contributing to beneficial left ventricle (LV) remodeling after acute myocardial infarction (AMI).

## Background

Cardiac stem cells antigen-1 positive (Sca-1+) cells possess properties of cardiac and endothelial cell differentiation (Oh et al. 2003; Matsuura et al. 2004; Takamiya et al. 2011) and occur in adult murine hearts, which contain potential stem cells (Wang et al. 2014; Tateishi et al. 2007). For example, the knockdown of Sca-1 transcripts in cardiac stem cells (CSCs) significantly inhibited CSC proliferation and survival, resulting in decreased myocardial contractility (Tateishi et al. 2007; Bailey et al. 2012). Conversely, the expression of Sca-1+ CSCs significantly increased in the mouse heart after acute myocardial infarction (AMI) (Wang et al. 2006). Hypoxic injury such as AMI promoted Sca-1+ CSC migration to the infarcted zone to induce myocardial renewal (Liu et al. 2013).

Sca-1+ CSCs account for only 2% of all heart cells (Oh et al. 2003). Limited numbers of Sca-1+ CSCs and their limited proliferative potential were the major limitations for use in various studies. However, various genetically engineered stem cells inserted with human telomerase reverse transcriptase (TERT) gene showed immortalization without chromosomal aberrations or malignant transformation (Huang et al. 2008; Burk et al. 2019; Wolbank et al. 2009). Our previous study revealed that genetically engineered CSCs (GECS) maintained the stemness even after long-term culture (Park et al. 2016) .

In transitional two-dimensional (2D) cell systems, there were limitations in viability, proliferation, differentiation, and function. However, spheroids provided a three-dimensional environment that enabled intensive cell-to-cell contact and enhanced regenerative properties (Laschke and Menger 2017). To form spheroids, poly-2-hydroxyethyl methacrylate (poly-HEMA) is typically used as a non-adherent coating material for cell aggregation (Long et al. 2014). In our previous study, sphere formation of adipose stem cells was successfully engineered using poly-HEMA (Kim et al. 2015). Compared with adherent adipose stem cells, sphere formation could reduce antiapoptotic marker expression and increase that of hypoxic and growth factors.

Previous studies showed that CSCs secreted various cytokines and chemokines (Wollert and Drexler 2010; Tran and Damaser 2015). However, reports regarding paracrine factors secreted by sphere GECS (S-GECS) and their cardioprotective roles have been limited until now. This study aimed to establish S-GECS, investigate paracrine factors secreted by the S-GECS, and clarify their cardioprotective roles in in vitro and in vivo models.

## Methods

### Generation of GECS and characterization

**Fig. 1:**
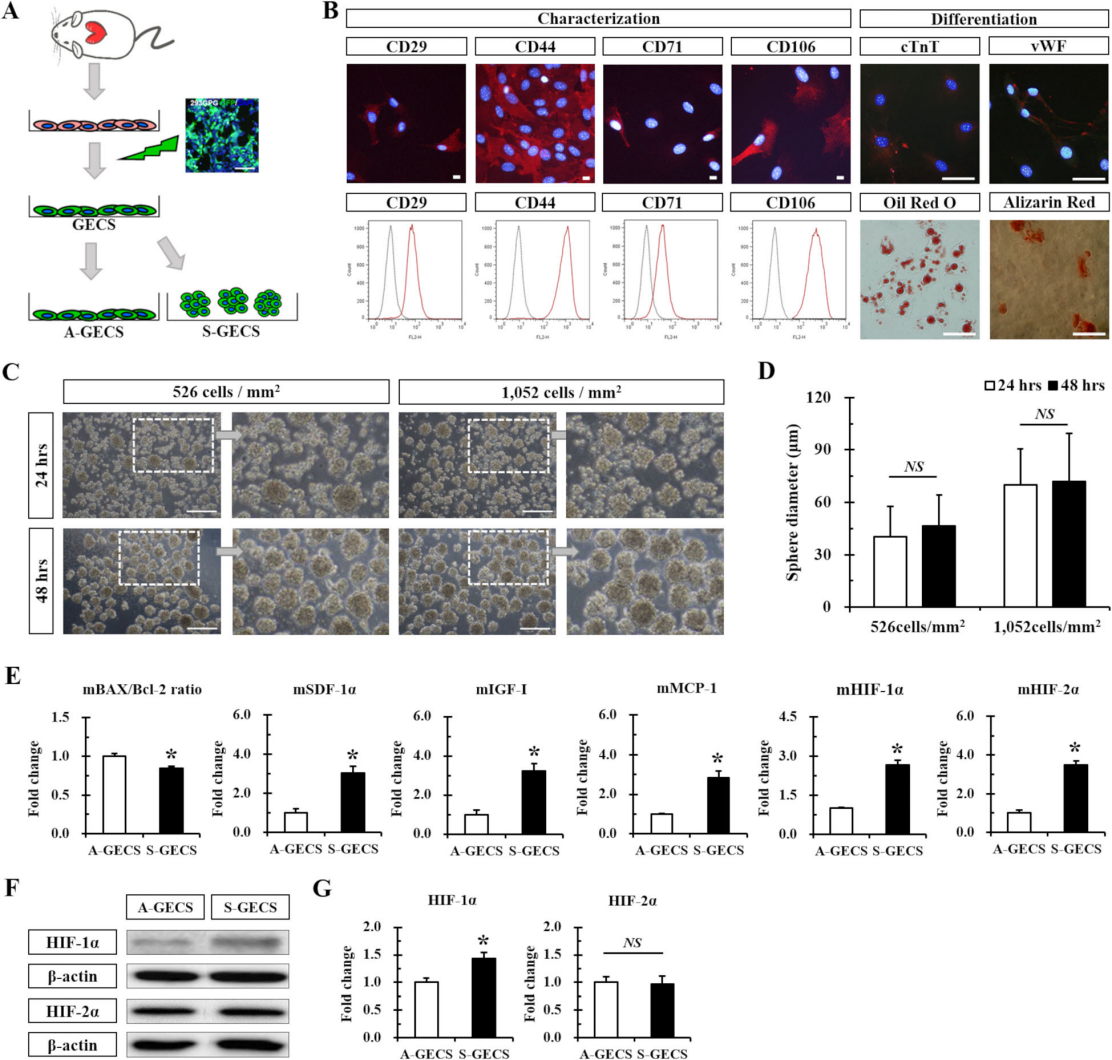
GECS generation and characterization. **a** Schematic diagram of GECS obtained from hTERT-immortalized Sca-1+ CSC lines. **b** Representative immunofluorescence images and flow cytometry of GECS. **c** Representative phase-contrast images of S-GECS produced on Poly-HEMA-coated plates 24 and 48 h after seeding. **d** Time-dependent increase in S-GECS diameter. *n* = 58 in each group. **e** Quantitative RT-PCR analysis of apoptotic, hypoxic, and growth factors in A-GECS and S-GECS for 48 h, each in triplicate. *p < 0.05 *vs.* A-GECS. **f** and **g** Western blot and quantification of HIF-1α and HIF-2α expression in A-GECS and S-GECS, each in triplicate. *p < 0.05 *vs.* A-GECS. All results are representative; scale bars represent 100 μm

GECS was obtained from hTERT-immortalized Sca-1+ CSC lines in our previous study (Fig. 1a) (Park et al. 2016). Sca-1+ CSCs sorted by magnetic-activated cell sorting were plated at 2 × 10^5^ cells in 6-cm culture dishes in Dulbecco’s modified Eagle’s medium-low glucose (DMEM-LG) supplemented with 10% fetal bovine serum (FBS) and 100 U/mL penicillin/streptomycin (P/S). Cells were infected with retroviruses harboring pLPCX-TERT-IRES-EGFP at 60% confluence for 3 days, and then selected in medium against 0.5 μg/mL puromycin in 10-cm culture dishes by repeated sub-culturing at a 1:3 ratio; this was performed three times per week for three weeks. For clonal analysis, the selected cells were plated in 96-well plates at one cell per 100 μL by limiting dilution in DMEM-LG supplemented with 10% FBS and 100 U/mL P/S. Briefly, wells containing one cell per well were selected by visual inspection alone 24 h after plating and then further cultured for 12 days. Among 20 single-cell-derived clones, two were finally selected based on microscopic examination of morphology, proliferation, green fluorescent protein expression, and hTERT expression.

### Phenotypic characterization of GECS by immunostaining

GECS was plated onto coverslips coated with 0.1% (w/v) gelatin in a 24-well plate. The cells were fixed with 4% paraformaldehyde (PFA; Sigma-Aldrich, St. Louis, MO, USA) in phosphate-buffered saline (PBS) for 10 min and washed with PBS + 0.1% Tween-20 (PBST). Cells were blocked for nonspecific binding by incubation in 5% normal goat serum (NGS; Invitrogen, Waltham, MA, USA) in PBST for 30 min. Next, cells were stained for 30 min with the following primary antibodies: CD14, CD29, CD31, CD44, CD45, CD71, CD90, CD106, CD117, Sca-1 (1:200 dilution; all from BD Biosciences, San Jose, CA, USA), CD34, and CD133 (1:200 dilution; both from e-Bioscience, San Diego, CA, USA). Cells were stained with Alexa Fluor 594-conjugated secondary antibodies (1:1000 dilution; Molecular Probes, Eugene, OR, USA) for 30 min and washed three times in PBST. Nuclei were stained with 4′,6-diamidino-2-phenylindole dihydrochloride (DAPI; Sigma-Aldrich, St. Louis, MO, USA), and cells were mounted using fluorescent mounting medium (DAKO, Carpinteria, CA, USA). Fluorescence images were obtained using a TE-FM Epi-Fluorescence System attached to an Olympus BX61 inverted microscope (Olympus, Tokyo, Japan).

### Phenotypic characterization of GECS by flow cytometry

GECS was fixed with 4% PFA in PBS for 10 min at room temperature (RT). The cells were subsequently incubated for 20 min at 4 °C with the following primary antibodies: CD14, CD29, CD31, CD34, CD44, CD45, CD71, CD90, CD106, CD117, CD133, and Sca-1 (1:200 dilution). After washing twice with PBS + 2% FBS, cells were incubated with fluorescein isothiocyanate (FITC)-conjugated goat anti-rat antibodies (1:1000 dilution; Sigma-Aldrich, St. Louis, MO, USA) for 15 min at 4 °C. For control experiments, the cells were stained with secondary antibodies only. After washing twice with PBS + 2% FBS, 30,000 cells per sample were analyzed on a FACS Calibur flow cytometer (BD Biosciences, San Jose, CA, USA). Data were analyzed using FlowJo v10 software.

### Differentiation potential of GECS

GECS was plated at a density of 1–2 × 10^4^ cells/mL in 24-well plates containing 0.1% (w/v) gelatin-coated glass coverslips. Cells were cultured in DMEM-LG supplemented with 10% FBS and 100 U/mL P/S for 2–3 days. Cardiomyogenic differentiation of GECS was induced by incubation in DMEM-LG supplemented with 10% FBS, 100 U/mL P/S, and 1 μM 5-azacytidine (Sigma-Aldrich, St. Louis, MO, USA) for 21 days. Cultures were maintained by media exchange every 3–4 days. Endothelial differentiation of GECS was induced by incubation in 60% DMEM-LG and 40% MCDB-201 (Sigma-Aldrich, St. Louis, MO, USA) supplemented with 1 × insulin-transferrin-selenium, 1 × linoleic acid-BSA, 10^− 8^ M dexamethasone, 10^− 4^ M ascorbic acid 2-phosphate (all from Sigma-Aldrich, St. Louis, MO, USA), 100 U/mL P/S, and 20 ng/mL vascular endothelial growth factor (VEGF; R&D Systems, Minneapolis, MN, USA) for 21 days. Cultures were maintained by media exchange every 3–4 days.

To assess cardiac or endothelial differentiation, the cells were fixed with 4% PFA in PBS for 10 min, washed with PBST, and permeabilized with 0.1% Triton X-100 in PBS for 30 min. Cells were washed with PBST and blocked for nonspecific binding by incubation in PBST with 5% NGS for 30 min. Then, cells were incubated overnight at 4 °C with the following primary antibodies: anti-cardiac troponin T (cTnT; Developmental Studies Hybridoma Bank, Iowa City, Iowa, USA) and anti-von Willebrand factor (vWF; DAKO, Carpinteria, CA, USA). After washing three times with PBST, the cells were stained with Alexa Fluor 594-conjugated secondary antibodies (Molecular Probes, Eugene, OR, USA) for 30 min and washed three times in PBST. Nuclei were stained with DAPI. The cells were mounted with fluorescent mounting medium. Fluorescence images were obtained with a TE-FM Epi-Fluorescence system attached to an inverted microscope.

Adipogenic differentiation of GECS was induced by incubation in DMEM-LG supplemented with 5% FBS, 100 U/mL P/S, 1 μM dexamethasone, 10 μg/mL insulin, 100 μM indomethacin, and 0.5 μM methyl-isobutylxanthin (all from Sigma-Aldrich, St. Louis, MO, USA) for 10 days. Culture media were changed every 3 days. Adipogenic differentiation was assessed on day 10 using Oil Red O (Sigma-Aldrich, St. Louis, MO, USA) stain to indicate intracellular lipid accumulation. The cells were fixed with 4% PFA in PBS for 20 min, washed with 60% isopropanol, and stained with 0.3% Oil Red O solution in 60% isopropanol for 10 min. After washing three times with water, cells were de-stained in 100% isopropanol for 15 min. Osteogenic differentiation of GECS was induced by incubation in culture medium with 1 μM dexamethasone, 10 mM glycerophosphate, and 50 μM ascorbic acid (all from Sigma-Aldrich, St. Louis, MO, USA) for 21 days. Osteogenic differentiation was determined by Alizarin Red S (Sigma-Aldrich, St. Louis, MO, USA) staining.

### Culture of sphere-genetically engineered cardiac stem cell formation

To produce S-GECS, 6-well tissue culture plates were coated with poly-HEMA (Sigma-Aldrich, St. Louis, MO, USA), which was dissolved in cell culture-tested ethanol at 12 mg/mL concentration (Merck Millipore, Burlington, MA, USA), and incubated at 40 °C overnight. The S-GECS was seeded into each plate at a density of 1 × 10^6^ cells/well and cultured up to 48 h in culture media to identify the ideal conditions for sphere formation. Phase-contrast images were obtained using the Leica DMI 3000B upright microscope (Leica, Wetzlar, Germany). S-GECS diameters were quantified using ImageJ v1.32 software (National Institutes of Health, Bethesda, MD, USA).

### Real-time PCR

The total RNA was extracted from dishes plated at 5 × 10^5^ cells/10 cm for A-GECS and 526 or 1052 cells/mm^2^ for S-GECS after 24 or 48 h culture periods using Trizol reagent (Invitrogen, Waltham, MA, USA). Total RNA concentrations were determined using a Nanodrop 1000 spectrophotometer (Thermo Fisher Scientific, Waltham, MA, USA). First-strand cDNA was synthesized from 0.5 μg DNase-treated total RNA using 0.5 μg random hexamers (Invitrogen, Waltham, MA, USA) and 200 U Moloney murine leukemia virus reverse transcriptase (Invitrogen, Waltham, MA, USA) at 37 °C for 60 min at a total volume of 20 μL. Real-time PCR was performed using a real-time PCR thermal cycler (Bio-Rad, Hercules, CA, USA). Each reaction contained 12.5 μL 2X SYBR Green PCR Mix (Bio-Rad, Hercules, CA, USA), 1.5 μL forward primer (5 μM), 1.5 μL reverse primer (5 μM), 5 μL cDNA at 1:10 dilution, and 4.5 μL H_2_O.

The primers used for real-time PCR were as follows: mouse hypoxia-inducible factors 1 alpha (mHIF-1α), mouse hypoxia-inducible factor 2 alpha (mHIF-2α), mouse BCL2-associated X (mBAX), mouse B-cell lymphoma 2 (mBcl-2), mouse stromal cell-derived factor-1 alpha (mSDF-1α), mouse VEGF (mVEGF), mouse insulin-like growth factor 1 (mIGF-Ι), mouse monocyte chemoattractant protein 1 (mMCP-1), mouse β-actin, rat C-X-C chemokine receptor type 4 (rCXCR4), and rat β-actin (Additional file 1: Table S1). Real-time PCR data were pooled from three independent experiments. Relative gene expression levels were quantified based on Ct and normalized to the reference gene, β-actin.

### Western blotting

Samples were solubilized in lysis buffer (Cell Signaling Technology, Danvers, MA, USA) containing 1 mM phenylmethylsulfonyl fluoride for 1 h at 4 °C. Cell lysates were subjected to sodium dodecyl sulfate polyacrylamide gel electrophoresis and transferred onto a polyvinylidene difluoride transfer membrane. The membranes were stained using the following primary antibodies: anti-HIF-1α, anti-HIF-2α (1:1000 dilution; both from R&D Systems, Minneapolis, MN, USA), anti-CXCR4 (1:1000 dilution; Abcam, Cambridge, UK), anti-Akt (1:1000 dilution; Santa Cruz Biotechnology, Dallas, TX, USA), anti-phospho-Akt, anti-extracellular signal-regulated kinases (Erk) 1/2, anti-phospho-Erk1/2 (1:1000 dilution; all from Cell Signaling Technology, Danvers, MA, USA), and anti-β-actin (1:5000 dilution; Sigma-Aldrich, St. Louis, MO, USA). The secondary antibodies used were anti-rabbit IgG-horseradish peroxidase (HRP), anti-mouse IgG-HRP (1:3000 dilution; both from Cell Signaling Technology, Danvers, MA, USA), and anti-goat IgG-HRP (1:2000 dilution; R&D Systems, Minneapolis, MN, USA). The membranes were exposed to the ChemiDoc™ Touch Imaging System (Bio-Rad, Hercules, CA, USA) using an enhanced chemiluminescence detection system. Signal intensity was analyzed with Quantity One software (Bio-Rad, Hercules, CA, USA).

### Cytokine/chemokine antibody array

The experiments were performed using the mouse cytokine/chemokine antibody array kit (RayBiotech, Peachtree Corners, GA, USA). Twenty one different cytokines were evaluated: cardiotrophin-1 (CT-1), MCP-1, MCP-5, Platelet factor 4 (PF4), basic fibroblast growth factor (b-FGF), epidermal growth factor (EGF), hepatocyte growth factor (HGF), IGF-1, IGF-2, granulocyte colony stimulating factor (G-CSF), macrophage-CSF (M-CSF), granulocyte-macrophage CSF (GM-CSF), stem cell factor (SCF), SDF-1α, VEGF-A, VEGF-D, intracellular adhesion molecule 1 (ICAM-1), vascular cell adhesion molecule 1 (VCAM-1), milk fat globule-EGF factor 8 (MFG-E8), thrombopoietin (TPO), tumor necrosis factor-α (TNF-α), galectin-1, and galectin-3. Cell lysates were prepared from A-GECS and S-GECS. The mouse cytokine/chemokine antibody array membranes were blocked for 1 h and then incubated with cell lysates, biotin-conjugated anti-cytokine antibodies, and HRP-conjugated streptavidin for 2 h each at room temperature. Finally, membranes were exposed to ChemiDoc™ using an enhanced chemiluminescence detection system. Blots were quantified using Quantity One software.

### Enzyme-linked immunosorbent assay (ELISA)

To evaluate SDF-1α, MCP-1, and IGF-1 levels secreted from GECS-conditioned medium (GECS CM), 1052 GECS cells/mm^2^ were plated on poly-HEMA-coated 6-well plates. After 48 h of culture, SDF-1α, MCP-1, and IGF-1 supernatant levels were determined by Mouse CXCL12/SDF-1α, Mouse CCL2/JE/MCP-1, and Mouse IGF-1 immunoassay kits (R&D Systems, Minneapolis, MN, USA). The ELISA reaction product was quantified by measuring absorbance at 450 nm and 540 nm using an iMark microplate reader, and data were analyzed using Microplate Manager v. 6.0 (both from Bio-Rad, Hercules, CA, USA).

### Apoptosis assay

To induce hypoxia via cobalt chloride (CoCl_2_; Sigma-Aldrich, St. Louis, MO, USA), the cardiomyoblast cell line H9c2 was used. Then, 2 × 10^5^ H9c2 cardiomyoblasts were seeded in six-well culture dishes and allowed to reach ~ 80% confluence in DMEM-LG supplemented with 10% FBS and 100 U/mL P/S in a humidified incubator at 37 °C and 5% CO_2_. Cells were treated with or without 150 μM CoCl_2_ for 24 h in Mesencult MSC Basal Medium (STEMCELL Technologies, Vancouver, Canada) supplemented with 2% FBS and 100 U/mL P/S or in S-GECS CM. Annexin V (AV) and propidium iodide (PI) staining were performed using an FITC Annexin V Apoptosis Detection Kit II (BD Biosciences, San Jose, CA, USA) according to the manufacturer’s instructions, analyzed on a FACS CantoII flow cytometer (BD Biosciences, San Jose, CA, USA). Data were analyzed using FlowJo v10 software.

### Transfection of CXCR4 siRNA into H9c2 cardiomyoblasts

H9c2 cells were cultured on 6-well plates at a density of 1.5 × 10^5^ cells and transfected with 60 nM rat CXCR4 siRNA candidate 1 forward (5′-CACAAGUGGAUCUCCAUCA − 3′) and reverse (5′- UGAUGGAGAUCCACUUGUG − 3′), rat CXCR4 siRNA candidate 2 forward (5′- GAGCAUUGCCAUGGAAAUA − 3′) and reverse (5′- UAUUUCCAUGGCAAUGCUC − 3′), rat CXCR4 siRNA candidate 3 forward (5′-CCAUGGCUGACUGGUACUU − 3′) and reverse (5′- AAGUACCAGUCAGCCAUGG − 3′), or negative control (NC) siRNA (all from Bioneer, Oakland, CA, USA) using Lipofectamine RNAiMAX (Invitrogen, Waltham, MA, USA) for 48 h as suggested by the manufacturer. Then, the cells were changed to a medium containing 10% FBS for use in subsequent experiments.

### Acute myocardial infarction model and cell transplantation

A total of 18 rats were sorted randomly at a 1: 1: 1 ratio into 3 groups: the sham control, A-GECS, and S-GECS. Female SD rats weighing 180–200 g were anesthetized by intraperitoneal (IP) injection with a mixture of ketamine (60 mg/kg) and xylazine hydrochloride (7.5 mg/kg). An 18-gauge angiocatheter (BD Biosciences, San Jose, CA, USA) was utilized as an intubation tube throughout the procedure. The left coronary artery of the heart was ligated with a 6–0 silk suture located 5 mm from the left coronary atrial appendage. After confirming the presence of AMI, A-GECS (1 × 10^6^) or S-GECS prepared in 100 μL culture medium were injected at three peri-infarct areas. S-GECS were harvested after 48 h of plating 1 × 10^6^ GECS/well on poly-HEMA-coated 6-well plates. Larger 25-gauge syringes were used to deliver S-GECS (Cho et al. 2017). The A-GECS seeding confluency was 90% (Dong et al. 2012). For the control treatment, 100 μL culture medium was injected. After cell transplantation, the chest wall, muscle layers, and skin were closed with 3–0 silk sutures. All AMI-induced rats were continuously monitored from surgery to recovery. For sacrifice, rats were euthanized by IP injection with a mixture of ketamine (60 mg/kg) and xylazine hydrochloride (7.5 mg/kg).

### Echocardiographic analysis

Echocardiography was performed at 1, 7, and 28 days after cell transplantation using a Vivid 7 Echocardiography System (GE Healthcare, Chicago, IL, USA) with a 10 MHz small linear array transducer for animal research. Parasternal long- and short-axis views were obtained. Rats were anesthetized with a mixture of ketamine (60 mg/kg) and xylazine hydrochloride (7.5 mg/kg). The posterior wall thickness in diastole and systole (PWTd and PWTs, *respectively*), left ventricular end of diastolic and systolic volumes (LVEDV and LVESV, *respectively*), and left ventricular anterior wall thickening were measured using a 2-dimensional M-mode view. The LV volume and ejection fraction (EF) were calculated using the modified Simpson’s method. The fractional shortening (FS) percentage was also computed. All parameters were assessed over 3 consecutive cardiac cycles, and each value was averaged from two measurements. Echocardiography was performed by an experienced cardiologist who was blind to the study group.

### Immunohistochemical analysis of tissue sections

The heart tissues were fixed in 4% PFA and embedded in paraffin. Then, 5 or 10 μm thick sections were divided from the infarcted LV wall and septum of paraffin-embedded hearts.

Masson’s trichrome (MT) staining was performed using the Trichrome Stain Kit (Sigma-Aldrich, St. Louis, MO, USA) with the following modifications: nuclei were stained with Celestine Blue solution followed by Gill’s hematoxylin stain (both from Sigma-Aldrich, St. Louis, MO, USA), and tissue was incubated for 1 h in Bouin’s solution before muscle staining with Biebrich scarlet-acid fuchsin (Sigma-Aldrich, St. Louis, MO, USA). Stains were quantified using Image-Pro 7.0 software (Media Cybernetics, Rockville, MD, USA).

Hematoxylin and eosin (H&E) staining was conducted by the conventional method. For immunohistochemical staining, the heart tissue sections were treated with proteinase K (Merck Millipore, Burlington, MA, USA) incubated, blocked with 5% NGS, and washed with PBST. Then, the tissue sections were incubated with primary antibodies against CXCR4 (Abcam, Cambridge, UK) and CD31 (BD Biosciences, San Jose, CA, USA). The tissue sections were subsequently stained with Alexa Fluor 594-conjugated anti-rabbit or anti-rat antibodies (all from Molecular Probes, Eugene, OR, USA) and then incubated with DAPI. Fluorescence images were obtained using a TE-FM Epi-Fluorescence System attached to an Olympus BX61 inverted microscope. CXCR4 cells were quantified using Image-Pro 7.0 software.

### Statistical analysis

All statistical values are expressed as the mean ± standard deviation (SD). Significant differences between means were determined using Student’s *t*-test or analysis of variance (ANOVA) followed by the Tukey test. Statistical significance was set at *p* < 0.05. All statistical analyses were performed using Sigma STAT software v4.0 (IBM, San Jose, CA, USA).

## Results

### Sphere formation of GECS by poly-HEMA

GECS phenotypic characterization was assessed by immunostaining and flow cytometry with different cell surface antibodies. We confirmed that CSCs were positive for CD29, CD44, CD71, CD106, and Sca-1 but negative for CD14, CD31, CD34, CD45, CD90, CD117, and CD133 using fluorescence immunostaining (Fig. 1b and Additional file 1: Figure S1A), and flow cytometry results were consistent with these findings (Fig. 1b and Additional file 1: Figure S1A). Multipotent differentiation was also identified (Fig. 1b).

S-GECS was successfully formed on poly-HEMA-coated plates after seeding. The sphere diameter increased along with culture time, and S-GECS morphological changes were observed with phase contrast imaging (Fig. 1c and d). Regarding the cell number per mm^2^, sphere diameter, and morphology via phase contrast imaging, the optimal conditions for S-GECS was considered to be 1052 GECS cells/mm^2^ and 48 h culture time (Fig. 1c). Total cell numbers in each sphere were higher in the sample with 1052 GECS cells/mm^2^ than in that with 526 GECS cells/mm^2^ at 48 h (165.65 ± 24.09 *vs.* 641.18 ± 60.47).

HIF-1α and HIF-2α, known as hypoxia-induced survival factors, showed significantly increased mRNA expression, which was increased 1.75-fold and 1.89-fold, *respectively*, after the 48 h culture compared to 24 h (Additional file 1: Figure S1B). To confirm this condition, qPCR was performed. The 48 h culture showed a similar ratio of the mRNA expression of pro-apoptotic factor BAX/antiapoptotic factor Bcl-2, which was 1.10-fold higher than that in the 24 h culture. To compare the expression of growth factors between the 24 and 48 h cultures, SDF-1α, VEGF-A, IGF-1, and MCP-1 were analyzed by qPCR. The 48 h S-GECS culture showed significantly higher mRNA expression of SDF-1α (2.02-fold), VEGF-A (1.30-fold), IGF-1 (1.4-fold), and MCP-1 (1.19-fold). SDF-1α and IGF-1 were significantly increased in the 48 h culture compared to the 24 h culture.

### Increased S-GECS survival and growth factors

S-GECS and A-GECS were cultured for 48 h to compare the levels of growth factors using qPCR. Compared to A-GECS, S-GECS showed a significantly decreased ratio of the mRNA expression of pro-apoptotic factor BAX/antiapoptotic factor Bcl-2 (0.84-fold) (Fig. 1e). To compare growth factor levels between S-GECS and A-GECS, SDF-1α, IGF-1, and MCP-1 were analyzed by qPCR. S-GECS showed significantly higher mRNA expression of SDF-1α (3.09-fold), IGF-1 (3.33-fold), and MCP-1 (2.72-fold) compared to A-GECS (Fig. 1e). To confirm the microenvironment derived from sphere formation, HIF-1α and HIF-2α showed significantly increased mRNA expression, which was 2.71-fold and 3.56-fold higher, *respectively*, in S-GECS than in A-GECS (Fig. 1e). In addition, western blot analysis of the 48 h S-GECS culture showed considerably increased HIF-1α expression (1.43-fold) compared to the A-GECS culture (Fig. 1f). However, HIF-2α expression levels were similar between the two groups.

**Fig. 2:**
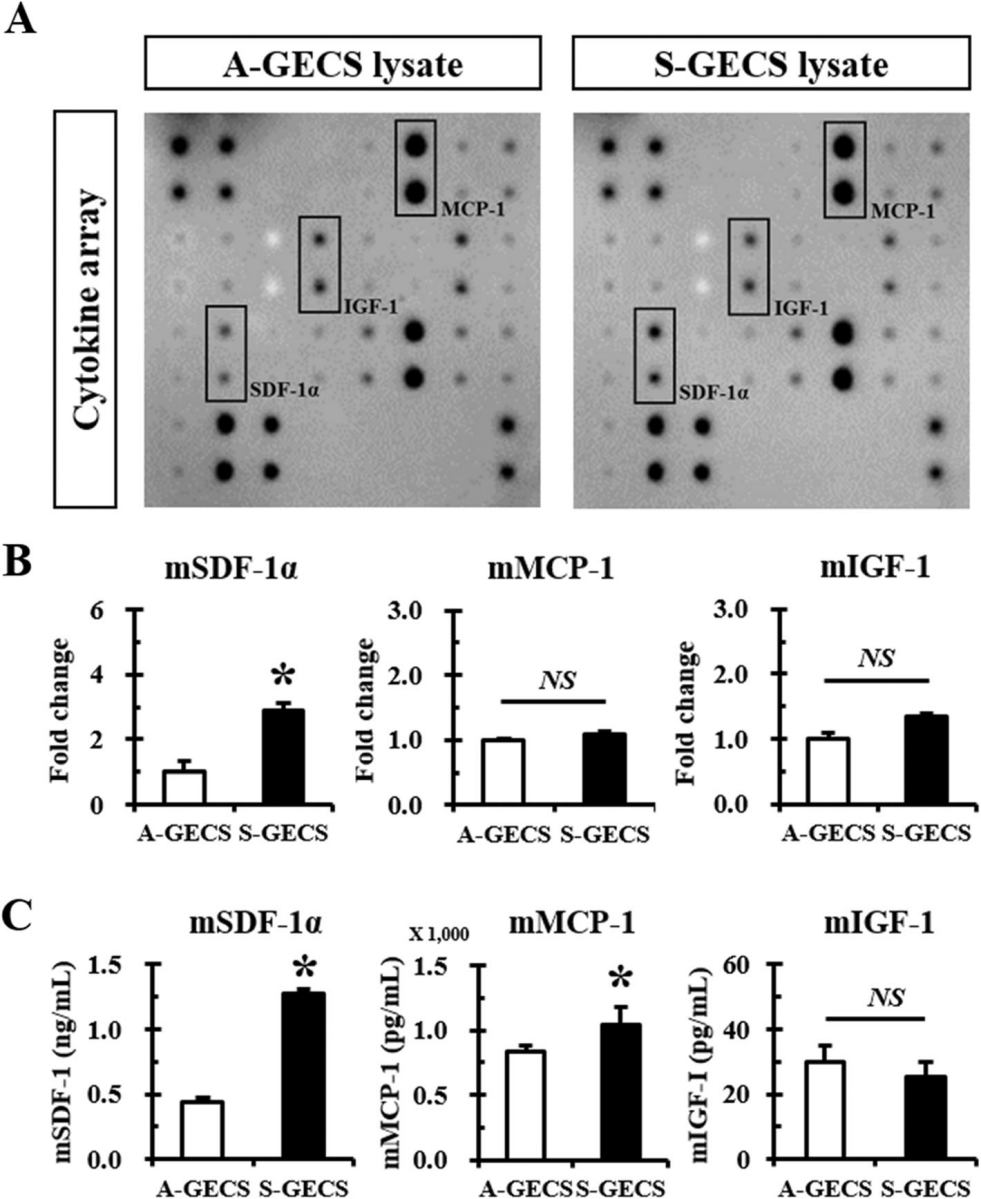
Cytokines/chemokine antibody array panels of S-GECS lysates. **a** Representative cytokine/chemokine antibody array panel of A-GECS and S-GECS lysates. **b** Comparison of highly expressed SDF-1α, MCP-1, and IGF-1 between A-GECS and S-GECS. Relative expression of cytokines and chemokines was measured by densitometry, each in triplicate. *p < 0.05 *vs.* A-GECS. (C) ELISA of SDF-1α, MCP-1, and IGF-1 between A-GECS and S-GECS, each in triplicate. *p < 0.05 *vs.* A-GECS

Furthermore, a mouse cytokine antibody array containing 21 growth factors and cytokines was performed to identify the paracrine factors secreted from S-GECS and A-GECS cell lysates (Fig. 2a). Cytokine array analysis showed different growth factor and cytokine expression profiles (Additional file 1: Figure S2). Compared with A-GECS, S-GECS showed quantitatively higher SDF-1α expression (Fig. 2a). Other cytokines, such as CT-1, HGF, G-CSF, and VEGF-A, were very weakly detected in the S-GECS lysate. Qualitative analysis showed increased expression of SDF-1α (2.91-fold), IGF-1 (1.34-fold), and MCP-1 (1.09-fold) in S-GECS (Fig. 2b). However, only SDF-1α was significantly increased in S-GECS compared to A-GECS.

To clarify which paracrine factors were secreted by A-GECS and S-GECS, growth factor expression was compared by ELISA (Fig. 2c). Compared with A-GECS and S-GECS showed quantitatively higher expression of SDF-1α (2.89-fold) and MCP-1 (1.24-fold), but no differences were found in IGF-1.

### S-GECS CM protects H9c2 cardiomyocytes from CoCl_2_-induced hypoxic injury

**Fig. 3:**
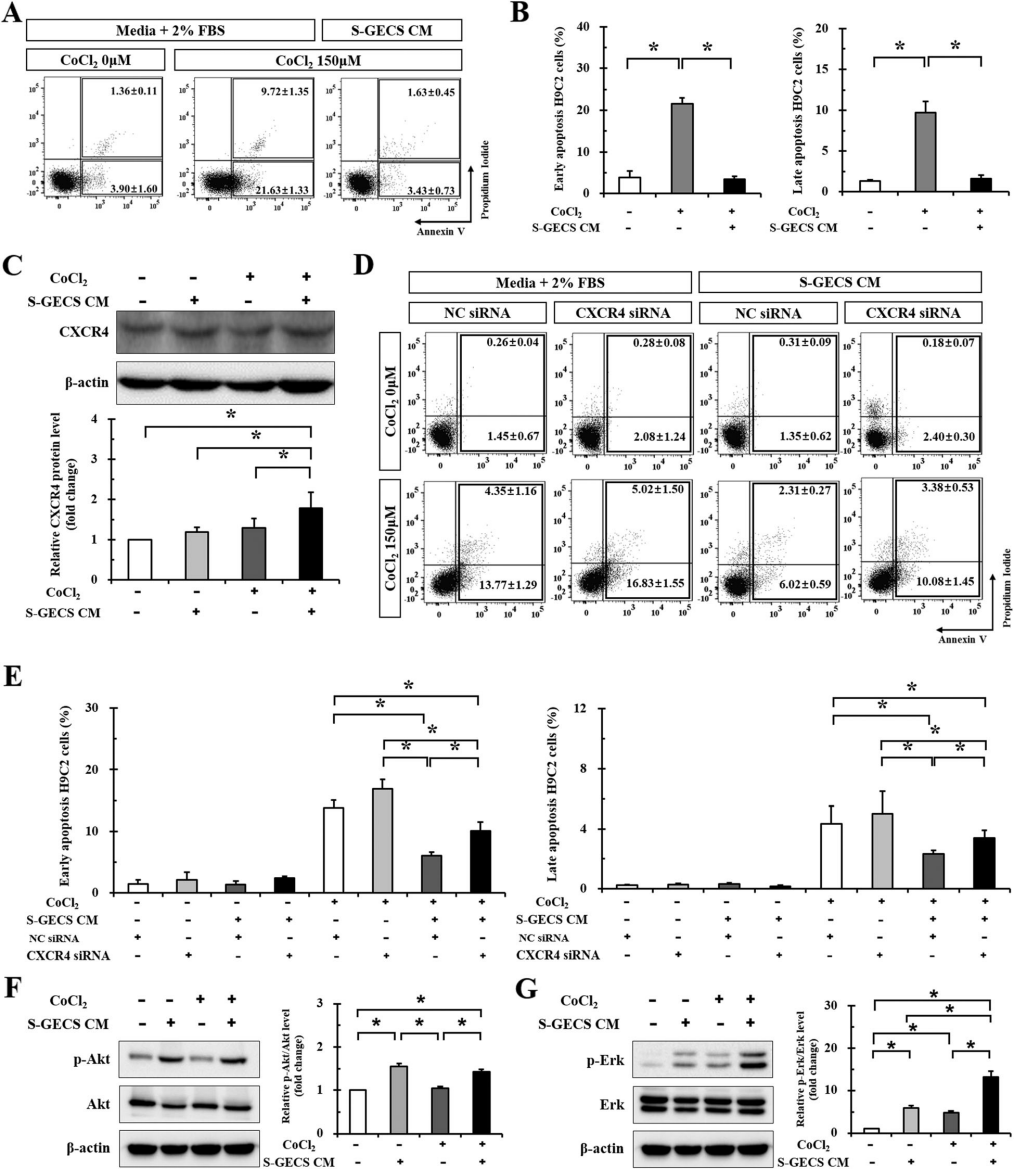
Cardioprotective effects by SDF-1α secreted from S-GECS in hypoxic injury. **a** The effect of S-GECS CM on early and late apoptosis of CoCl_2_-treated cardiomyoblasts. **b** Changes in the proportion of early apoptotic (AV+/PI-) cardiomyoblasts during CoCl_2_-induced hypoxic injury after S-GECS CM treatment, each in triplicate. *p < 0.05. **c** Western blot analysis of CXCR4 expression in H9c2 cardiomyoblasts treated with CoCl_2_ for 24 h, each in quintuplicate. *p < 0.05. **d** The effect of S-GECS CM and gene silencing via CXCR4 siRNA CM on early and late apopotosis of cardiomyoblasts with and without CoCl_2_ treatment. **e** Comparisons of the proportion of early apoptotic (AV+/PI-) cardiomyoblasts with and without CXCR4 siRNA before and after S-GECS CM treatment, each in triplicate. *p < 0.05. **f** Western blot of Akt signaling with and without CoCl_2_ and S-GECS CM treatment, each in triplicate. *p < 0.05. **g** Western blot of Erk signaling with and without CoCl_2_ and S-GECS CM treatment, each in triplicate. *p < 0.05. Data represent the mean ± SD

CoCl_2_ treatment induced apoptotic death of cardiomyocytes through HIF-1α-dependent stabilization of p53 protein. Therefore, CoCl_2_ was used to produce a hypoxia-mimicking environment and to determine whether paracrine factors secreted from S-GECS protect against CoCl_2_-induced cardiomyocyte death. The effect of S-GECS CM on early and late apoptosis in CoCl_2_-treated cardiomyocytes is shown in Fig. 3a. At the baseline, the rates of early and late apoptosis were 3.90% and 1.36%, *respectively*. S-GECS CM significantly reduced the proportion of early apoptotic (AV+/PI-) cardiomyocytes during CoCl_2_-induced hypoxic injury from 21.63 to 3.43% (Fig. 3b); furthermore, the percentage of late apoptotic (AV+/PI+) cardiomyocytes was also reduced from 9.72 to 1.63% in the same conditions.

### Cardioprotection by SDF-1α secreted from S-GECS

Since SDF-1α was significantly increased in various analyses, CXCR4 expression was investigated. Western blot analysis showed that CXCR4 expression was significantly increased in H9c2 cardiomyocytes treated with CoCl_2_ for 24 h (1.30-fold) as well as in those treated with S-GECS CM (1.20-fold). Co-treatment with CoCl_2_ and S-GECS CM showed the highest expression of CXCR4 (1.78-fold) (Fig. 3c).

Gene silencing via CXCR4 siRNA was performed to investigate whether the protective effects of S-GECS was abolished (Fig. 3d). The percentage of apoptosis in H9c2 cardiomyocytes treated with CoCl_2_ and NC siRNA for 24 h was significantly decreased from 13.77 ± 1.29% to 6.02 ± 0.59% in the presence of S-GECS CM (Fig. 3e). In H9c2 cardiomyocytes treated with CoCl_2_ and CXCR4 siRNA for 24 h, the presence of S-GECS CM significantly decreased the percentage of apoptosis from 16.83 ± 1.55% to 10.08 ± 1.45%. In addition, the percentage of apoptosis was significantly increased after treatment with CXCR4 siRNA compared to NC siRNA in CoCl_2_- and S-GECS CM-treated cells (6.02 ± 0.59% *vs.* 10.08 ± 1.45%, *p* < 0.05).

To investigate the downstream pathway of SDF-1α/CXCR4, western blot analysis of the Akt and Erk signaling pathways was performed. Interestingly, the p-Akt level did not increase with CoCl_2_ treatment (Fig. 3f), but was significantly increased following S-GECS CM treatment (1.55-fold). Furthermore, CoCl_2_ and S-GECS CM treatment significantly increased p-Erk levels (4.84-fold and 5.93-fold, *respectively*) compared with non-treated condition (Fig. 3g). The co-treatment of CoCl_2_ and S-GECS CM increased the p-Erk level up to 13.11-fold.

### Cardioprotection after S-GECS transplantation into infarcted myocardium

**Fig. 4:**
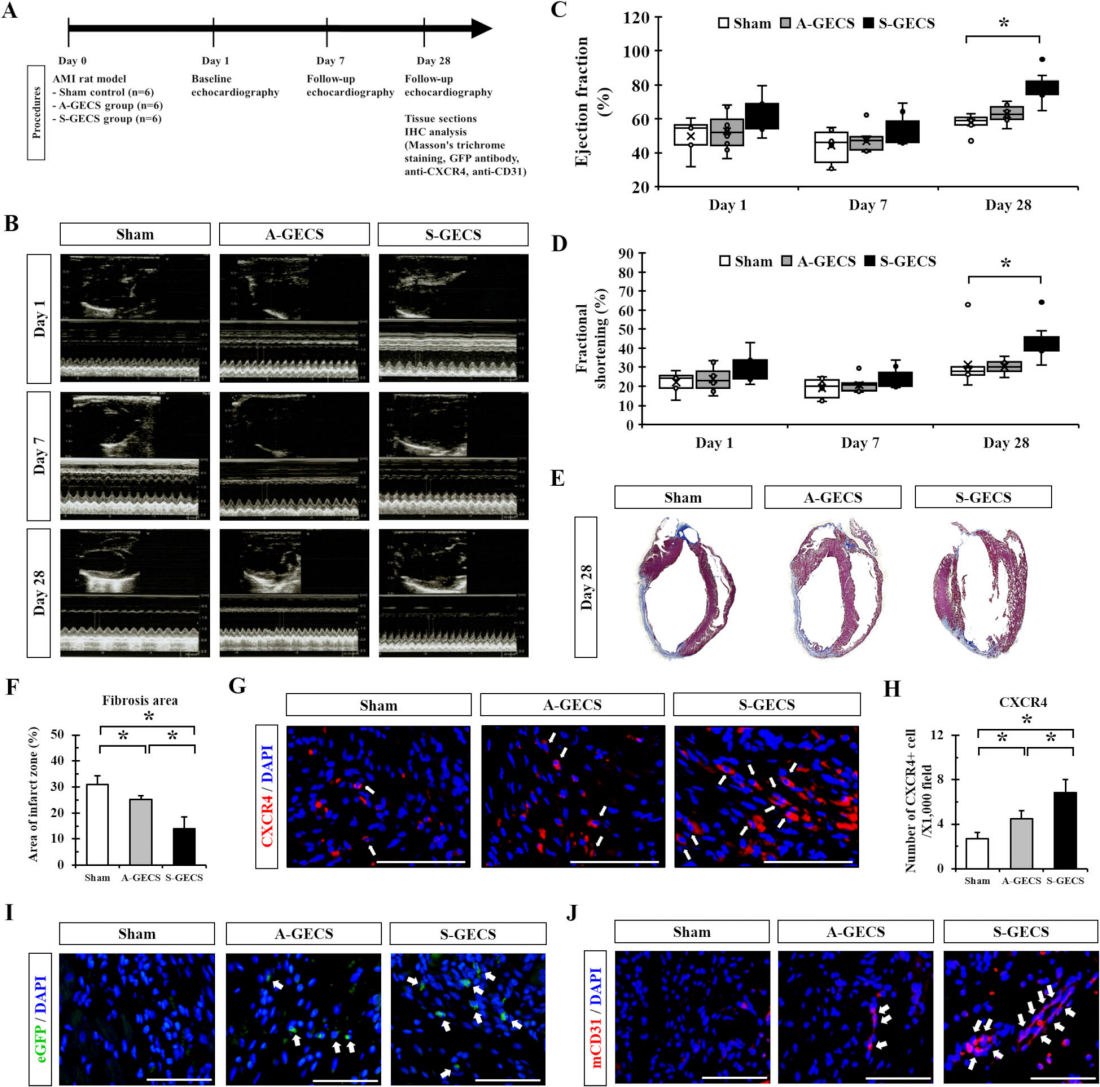
S-GECS transplantation into infarcted myocardium and cardiac regeneration. **a** Schematic diagram of S-GECS transplantation into infarcted myocardium. **b** Representative echocardiographic images at 1, 7, and 28 days following cell transplantation. **c** and **d** Cardiac function assessed by EF and FS at 1, 7, and 28 days following cell transplantation among three groups. *n* = 4, 5, and 5 rats in each group, *p < 0.05. **e** and **f** Masson’s trichrome staining on tissue sections after transplantation to determine the degree of fibrosis in A-GECS and S-GECS groups. *p < 0.05. **g** and **h** CXCR4 expression in the border zone in the S-GECS group. *p < 0.05. Nuclei were stained with DAPI (blue). **i** Representative images showing eGFP-positive transplanted cells in the border zone at 28 days of transplantation. Nuclei were stained with DAPI (blue). **j** Endothelial marker mCD31 expression in the border zone in the S-GECS group. Nuclei were stained with DAPI (blue). All quantification analyzed using images from *n* = 4, 5, and 5 rats in each group, each measured in triplicate. All results are representative; scale bars represent 100 μm

S-GECS was injected into the peri-infarct area of a rat model with AMI. Rats in the sham group were injected with an equivalent volume of medium with no cells for use as a control (Fig. 4a).

Cardiac function was assessed by echocardiography at 1, 7, and 28 days following transplantation (Fig. 4b). The baseline EF was similar among the sham, A-GECS, and S-GECS groups (52.93 ± 5.03%, 52.95 ± 10.34%, and 58.65 ± 7.62%, *respectively*; *p* = 0.481) (Table 1 and Fig. 4c). The baseline FS was also similar among groups (Fig. 4d). At 28 days follow-up, S-GECS injection significantly improved EF compared to the sham or A-GECS groups (77.91 ± 4.98%, 59.20 ± 2.15%, and 63.85 ± 3.48%, *respectively*; *p* = 0.001) (Fig. 4c). EF was also significantly improved in the A-GECS group compared to the sham group (63.85 ± 3.48% *vs.* 59.20 ± 2.15%, *p* = 0.045). S-GECS increased the EF changes from days 1 to 28 compared to sham (19.26 ± 4.34% *vs.* 6.27 ± 3.10%, *p* = 0.001) or A-GECS groups (19.26 ± 4.34% *vs.* 10.90 ± 9.01%, *respectively*; *p* = 0.113). FS was also significantly improved in the S-GECS group. Changes from day 1 to 28 showed significant improvement among the three groups (*p* = 0.027). Changes in FS were significantly increased in the S-GECS group compared to the sham or A-GECS groups (15.11 ± 2.45% *vs.* 4.15 ± 1.53% or 7.09 ± 5.22%, *respectively*; *p* = 0.022 or *p* < 0.001).

**Table 1:** Changes in echocardiographic findings during the study

Days	Variables	Sham (*n* = 4)	Adherent CSCs (*n* = 5)	Sphere CSCs (*n* = 5)
Day 1	IVSd (cm)	0.11 ± 0.04	0.10 ± 0.02	0.08 ± 0.02
	LVIDd (cm)	0.69 ± 0.06	0.61 ± 0.05	0.51 ± 0.11
	LVPWd (cm)	0.15 ± 0.02	0.13 ± 0.04	0.09 ± 0.04
	LVIDs (cm)	0.53 ± 0.06	0.46 ± 0.05	0.37 ± 0.08
	EDV (ml)	0.76 ± 0.18	0.53 ± 0.12	0.35 ± 0.19
	ESV (ml)	0.36 ± 0.11	0.26 ± 0.09	0.14 ± 0.08
	EF (%)	52.93 ± 5.03	52.95 ± 10.34	58.65 ± 7.62
	FS (%)	23.67 ± 2.77	23.83 ± 6.20	26.90 ± 4.82
	SV (ml)	0.40 ± 0.09	0.28 ± 0.08	0.21 ± 0.12
	LVd mass (g)	1.03 ± 0.12	0.91 ± 0.08	0.78 ± 0.13
	RWT	0.44 ± 0.09	0.41 ± 0.11	0.36 ± 0.14
Day 7	IVSd (cm)	0.09 ± 0.02	0.08 ± 0.01	0.09 ± 0.01
	LVIDd (cm)	0.81 ± 0.05	0.83 ± 0.04	0.67 ± 0.14
	LVPWd (cm)	0.14 ± 0.03	0.13 ± 0.02	0.12 ± 0.03
	LVIDs (cm)	0.67 ± 0.06	0.66 ± 0.06	0.52 ± 0.13
	EDV (ml)	0.90 ± 0.41	1.25 ± 0.16	0.74 ± 0.37
	ESV (ml)	0.69 ± 0.17	0.67 ± 0.16	0.37 ± 0.19
	EF (%)	40.22 ± 11.62	46.79 ± 9.14	52.09 ± 9.90
	FS (%)	17.24 ± 5.90	20.64 ± 5.22	23.40 ± 5.96
	SV (ml)	0.46 ± 0.17	0.58 ± 0.11	0.37 ± 0.18
	LVd mass (g)	1.10 ± 0.11	1.07 ± 0.10	0.92 ± 0.12
	RWT	0.32 ± 0.12	0.31 ± 0.03	0.36 ± 0.13
Day 28	IVSd (cm)	0.09 ± 0.03	0.09 ± 0.02	0.11 ± 0.07
	LVIDd (cm)	0.93 ± 0.02	0.92 ± 0.07	0.80 ± 0.08
	LVPWd (cm)	0.14 ± 0.04	0.15 ± 0.03	0.18 ± 0.04
	LVIDs (cm)	0.67 ± 0.02	0.64 ± 0.06	0.46 ± 0.07
	EDV (ml)	1.70 ± 0.10	1.66 ± 0.32	1.15 ± 0.32
	ESV (ml)	0.70 ± 0.05	0.63 ± 0.19	0.26 ± 0.10
	EF (%)	59.20 ± 2.15	63.85 ± 3.48	77.91 ± 4.98
	FS (%)	27.82 ± 1.36	30.91 ± 2.33	42.02 ± 4.82
	SV (ml)	1.01 ± 0.08	1.06 ± 0.19	0.89 ± 0.25
	LVd mass (g)	0.98 ± 0.41	1.25 ± 0.19	1.24 ± 0.21
	RWT	0.29 ± 0.09	0.32 ± 0.05	0.45 ± 0.15

*CSC* Cardiac stem cell, *IVDSd* Interventricular septal end diastole, *LVIDd* Left ventricular internal diameter end diastole, *LVPWd* Left ventricular posterior wall end diastole, *LVIDs*, Left ventricular internal diameter end systole, *EDV* End-diastolic volume, *ESV* End-systolic volume, *EF* Ejection fraction, *FS* fractional shortening, *SV* Systolic volume, *LVd mass* Left ventricular mass at end diastole, *RWT* Relative wall thickness

To explore the degree of fibrosis following transplantation, MT staining was performed (Fig. 4e and f). The fibrosis area was significantly decreased in the S-GECS group compared to the sham or A-GECS groups (13.94 ± 4.52% *vs.* 31.06 ± 3.23% or 25.20 ± 1.35%, *respectively*; *p* < 0.05) (Fig. 4f).

In the S-GECS group, CXCR4 expression in the border zone was significantly increased compared to that in the sham or A-GECS groups (6.83 ± 1.17 *vs.* 2.67 ± 0.58 or 4.50 ± 0.71 cells/× 1000 field, *respectively*; *p* < 0.05) (Fig. 4g and h). As GECS were tagged with eGFP, we detected eGFP signals after 28 days of transplantation to reveal their retention in the transplanted sites. The S-GECS group showed better cellular engraftment in the border zone than the A-GECS group (Fig. 4i). In addition, the expression of mCD31, which is known as an endothelial marker, was increased in the border zone compared to the sham or A-GECS groups (Fig. 4j).

## Discussion

This study showed that poly-HEMA could produce unattached and floating S-GECS. S-GECS showed significantly reduced antiapoptotic marker levels and increased levels of hypoxic and growth factors compared with A-GECS. These cardioprotective effects were demonstrated to be related with the CXCR4 and SDF-1α pathways. S-GECS transplantation into infarcted hearts could reduce the infarct size and improve cardiac function.

Cell therapy using stem cells remains a highlighted option for neovascularization in many cardiovascular ischemic diseases (Leeper et al. 2010). Among several stem cell types, cardiac stem cells have been investigated and established for use in myocardial regeneration (Beltrami et al. 2003). However, Sca-1+ CSCs account for only 2% of all heart cells, limiting their use for in vitro and in vivo studies. In the previous study, Sca-1+ CSCs were immortalized by the hTERT gene to reduce genetic unsteadiness and sustain similar phenotypic characteristics and multi-differentiation (Park et al. 2016). TERT activity is associated with stem cell function (Blasco 2007). TERT gene insertion into stem cells resulted in immortalization with no evidence of malignant transformation (Bentzon et al. 2005). The established Sca-1+ CSCs possessed a long-term proliferation capacity and multipotent differentiation potential. In addition, cardioprotective effects against hypoxic injury were identified.

Nevertheless, the 2D environment of the cell culture is another limiting factor. In 2D cultures, essential signaling pathways may be lost or compromised (Debnath and Brugge 2005). Cell morphology, receptor expression, or ECM interactions may also differ. As a result, 3D culture systems have been increasingly adopted. One relatively simple method for obtaining 3D spheroids is to generate forced-floating cells using poly-HEMA (Lin and Chang 2008). Poly-HEMA is neutrally charged and interferes with cell adhesion proteins, including integrins and cadherins, thereby contributing to the formation of spheres. In our study, sphere formation with poly-HEMA was successful, and this method was beneficial in various ways. The process was simple and reproducible, and equal cell numbers could be seeded in each well. Spheroid size was adjustable if needed. In addition, morphologically homogenous spheroids could be easily produced in large quantities. These morphologic characteristics corresponded to their proliferative potential and differentiation (Kern et al. 2006) as well as the survivability and behavior of cardiac stem cells in ischemic conditions (Li et al. 2007). Therefore, hypoxic factors and growth factors were significantly increased in S-GECS compared to A-GECS under the optimal conditions of 1052 GECS cells/mm^2^ and a 48 h culture period. As shown in Fig. 4i, the S-GECS group showed better cellular engraftment in infarcted regions than the A-GECS group. Increased cellular retention in the S-GECS group induced the improvement of cardiac function via secreting more SDF-1α compared with the A-GECS group. In accordance with our results, (Cho et al. 2013; Cho et al. 2012) reported that transplantation of cardiospheres to AMI animal models increased engraftment, differentiation, and paracrine effects in vivo compared with the same cells cultured in a 2D environment.

Although all cardiac stem cells expressing c-kit, MDR1, Sca-1, Flk-1, or islet-1 have growth potential, the patterns of secreted growth factors differ slightly (Barile et al. 2007). Particularly, cardiac Sca-1+ cells were enriched with various growth factors and cytokines that might be involved in cardiac repair (Oh et al. 2003). Although the cardioprotective effects of CSCs were evidenced by MCP-1, these effects were confirmed to be related with the CXCR4 and SDF-1α pathways in our study. SDF-1α was expressed in cardiomyocytes and fibroblasts and upregulated in myocardial ischemia (Hu et al. 2007). These results were consistent with previous findings that SDF-1α plays an important role in CSC migration, proliferation, and differentiation (Abbott et al. 2004). As a downstream pathway of SDF-1α, PI3K/Akt signaling was related with cell growth, survival, and protein synthesis (Cain and Ridley 2009). Similarly, the MEK/Erk pathway could transduce extracellular information into intracellular responses, owing to cell chemotactic responses (Tarcic et al. 2012). In fact, SDF-1α stimulated both p-Erk and p-Akt levels (Chen et al. 2015). These findings were consistent with our results that S-GECS treatment increased the levels of p-Erk and p-Akt via the SDF-1α/CXCR4 pathway. These secreted factors from S-GECS would be cardioprotective due to a paracrine mechanism. Hypoxic stress increased the production of various factors (Kinnaird et al. 2004). In fact, the ERK pathway was up-regulated in hypoxic conditions (Minet et al. 2000). As shown in our study, hypoxic injury induced by CoCl_2_ treatment increased only the level of p-Erk. The administration of S-GECS CM was able to induce beneficial effects even after hypoxic injury, which strongly suggests the involvement of the paracrine mechanism.

After S-GECS was injected into the infarcted area, infarct size was observed to decrease and cardiac function improved compared to controls. Cardiac function evaluated by EF and FS showed significant improvement in the S-GECS group compared to A-GECS or sham groups at 28 days follow-up. The fibrotic area determined by Masson’s trichrome staining showed consistent results due to CXCR4 and mCD31 expression in the border zone. The paracrine factors might influence nearby cells and decrease inflammation and fibrosis after AMI by promoting cardiac regeneration (Gnecchi et al. 2008).

There were some limitations in this study. Because this study confirmed significant cardioprotective effects via sphere formation using CSCs, further experiments using other stem cell types will be necessary. In addition, although our study investigated the downstream pathways of CXCR4 and SDF-1α, further investigations should be conducted to confirm the exact mechanisms of anti-apoptotic effects. Because functional improvements were assessed by echocardiography, the results may be subjective. Finally, xenograft transplantation was performed by mCSC implantation into rat models with AMI in our study. However, mesenchymal stem cells, including CSCs, have been reported to have low immunogenicity and antigen presentation capabilities. MSCs could moderate T-cell mediated immunological responses and assist cell homing to the ischemic site, thereby inducing immune tolerance to suppress rejection response to xenograft transplantation (Yagi et al. 2010; Potian et al. 2003). Future study should be investigated regarding recipient immune responses after CSC transplantation into AMI models.

## Conclusions

In this study, poly-HEMA was capable of producing unattached and floating S-GECS. S-GECS showed significantly reduced antiapoptotic marker levels and increased levels of hypoxic and growth factors compared with A-GECS. These cardioprotective effects were confirmed to be related with the CXCR4 and SDF-1α pathways. S-GECS transplantation into infarcted hearts could reduce the infarct size and improve cardiac function. Our results suggest that the transplanted S-GECS may possess cardioprotective roles in the infarcted myocardium due to paracrine effects, thereby contributing to the improvement of cardiac functions after AMI.

## Supplementary information

**Additional file 1:** Table S1. Primers used for real-time PCR in this study. Figure S1. Characterization of GECS. (A) Representative immunofluorescence images and flow cytometry of CSCs positive for CD29, CD44, CD71, CD106, and Sca-1. All results are representative; scale bars represent 100 μm. (B) Quantitative RT-PCR analysis of apoptotic, hypoxic, and growth factors in S-GECS for 24 and 48 h, each in quadruplicate. *p < 0.05 *vs.* 24 h. Figure S2. Mouse cytokines/chemokines antibody array panels of A-GECS and S-GECS lysates.

## Data Availability

All data generated or analyzed during this study are included in this published article and its supplementary information files. The datasets generated during the current study are available from the corresponding author on reasonable request.

## Abbreviations

A-GECS: Adherent-genetically engineered cardiac stem cells
AMI: Acute myocardial infarction
CM: Conditioned medium
CSC: Cardiac stem cell
GECS: Genetically engineered cardiac stem cells
Poly-HEMA: Poly-2-hydroxyethyl methacrylate
S-GECS: Sphere-genetically engineered cardiac stem cells
TERT: Telomerase reverse transcriptase
