# Supplementary material for: Cardioprotective effects of genetically engineered cardiac stem cells by spheroid formation on ischemic cardiomyocytes
Source: Mol Med. 2020 Jan 31;26:15. doi: 10.1186/s10020-019-0128-8 (PMC6995053; doi:10.1186/s10020-019-0128-8)
Supplement: Supplementary file 1 — Additional file 1: Table S1. Primers used for real-time PCR in this study. Figure S1. Characterization of GECS. (A) Representative immunofluorescence images and flow cytometry of CSCs positive for CD29, CD44, CD71, CD106, and Sca-1. All results are representative; scale bars represent 100 μm. (B) Quantitative RT-PCR analysis of apoptotic, hypoxic, and growth factors in S-GECS for 24 and 48 h, each in quadruplicate. *p < 0.05 vs. 24 h. Figure S2. Mouse cytokines/chemokines antibody array panels of A-GECS and S-GECS lysates. [file 10020_2019_128_MOESM1_ESM.docx]

**Supplementary Information**

**Cardioprotective Effects of Genetically Engineered Cardiac Stem Cell by Spheroid Formation on Ischemic Cardiomyocyte**

***Brief Title: Cardioprotection of Sphere Cardiac Stem Cell***

Han Saem Jeong^1,†^, Chi-Yeon Park^1,†^, Jong-Ho Kim^1^, Hyung Joon Joo^1^, Seung-Cheol Choi^1^, Ji-Hyun Choi^1^, I-Rang Lim^1^, Jae Hyoung Park^1^, Soon Jun Hong^1^, Do-Sun Lim^1,^*

^1^Department of Cardiology, Cardiovascular Center, Korea University Anam Hospital, Seoul 02841, Republic of Korea

^†^ These authors contributed equally to this work.

Contents:

Supplementary Table (Supplementary Table S1)

Supplementary Figure and Figure Legend (Supplementary Figure 1 and 2)

**Supplementary Table**

**Supplementary Table S1**. Primers used for real-time PCR in this study.

| Genes | Primer sequences (5’-3’) | | Size  (bp) |
| --- | --- | --- | --- |
|  | Forward | Reverse |  |
| mHIF-1α | GCACTAGACAAAGTTCACCTGAGA | CGCTATCCACATCAAAGCAA | 105 |
| mHIF-2α | GGGAACACTACACCCAGTGC | CAAGGGATTCTCCAAGGATG | 90 |
| mBAX | CGGCGAATTGGAGATGAACTG | GCAAAGTAGAAGAGGGCAACC | 160 |
| mBcl-2 | CGTAGCAGTCATCCTTTTTAGGAA | AAAGCAGCTTGCTAAATGCAG | 150 |
| mSDF-1α | TCTTGCTGTCCAGCTCTGC | CAGGCTGACTGGTTTACCG | 126 |
| mVEGF | ACCCTGGTGGACATCTTCCA | TCATCGTTACAGCAGCCTGC | 101 |
| mIGF-I | AGAAGCGATGGGGAAAATC | GAAGACGACATGATGTGTATCTTTATC | 96 |
| mMCP-1 | GGTGATCGCAACCCTAGC | TGTGTCGGCTGGATAGGC | 111 |
| mβ-actin | CTGTCCCTGTATGCCTCTG | ATGTCACGCACGATTTCC | 218 |
| rCXCR4 | GCCATGGCTGACTGGTACTT | GATGAAGGCCAGGATGAGAA | 99 |
| rβ-actin | AAGGCCAACCGTGAAAAGAT | ACCAGAGGCATACAGGGACA | 102 |

**Supplementary Figure and Figure Legend**


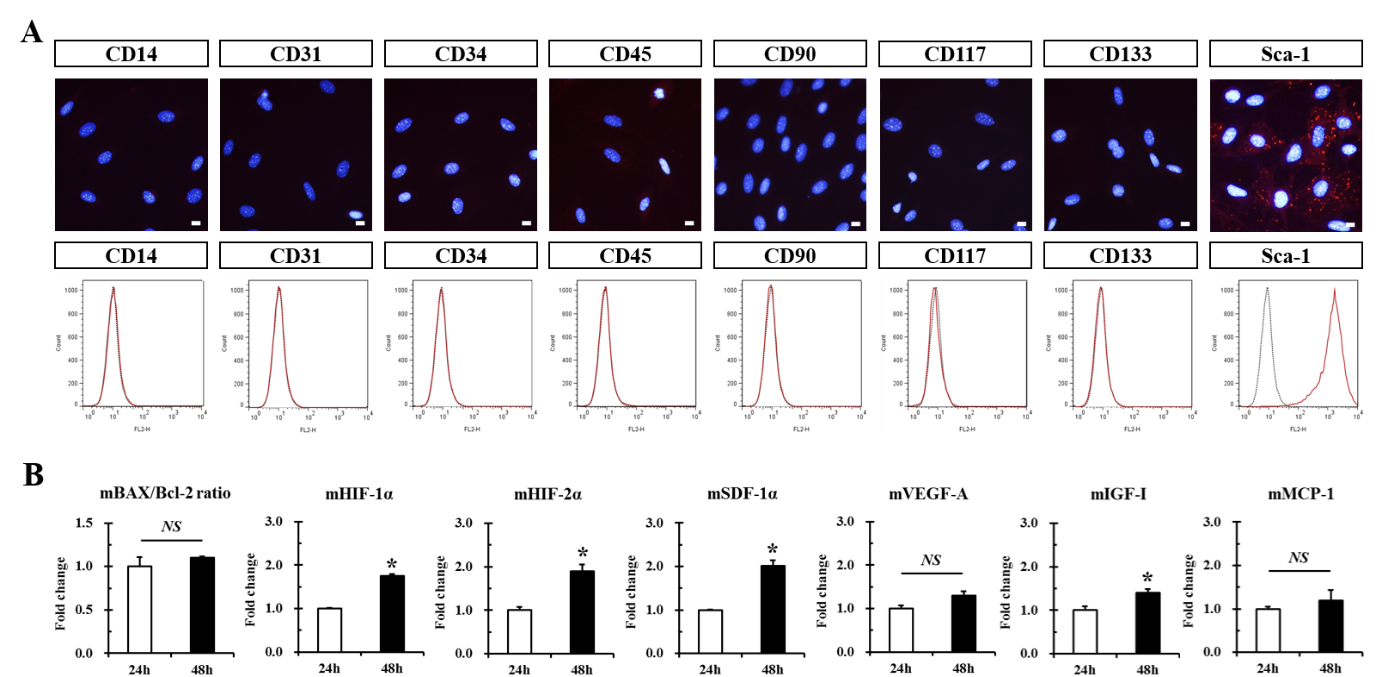


**Supplementary Figure S1.** Characterization of GECS. (A) Representative immunofluorescence images and flow cytometry of CSCs positive for CD29, CD44, CD71, CD106, and Sca-1. All results are representative; scale bars represent 100 μm. (B) Quantitative RT-PCR analysis of apoptotic, hypoxic, and growth factors in S-GECS for 24 and 48 hours, each in quadruplicate. **p* < 0.05 *vs.* 24 hours.


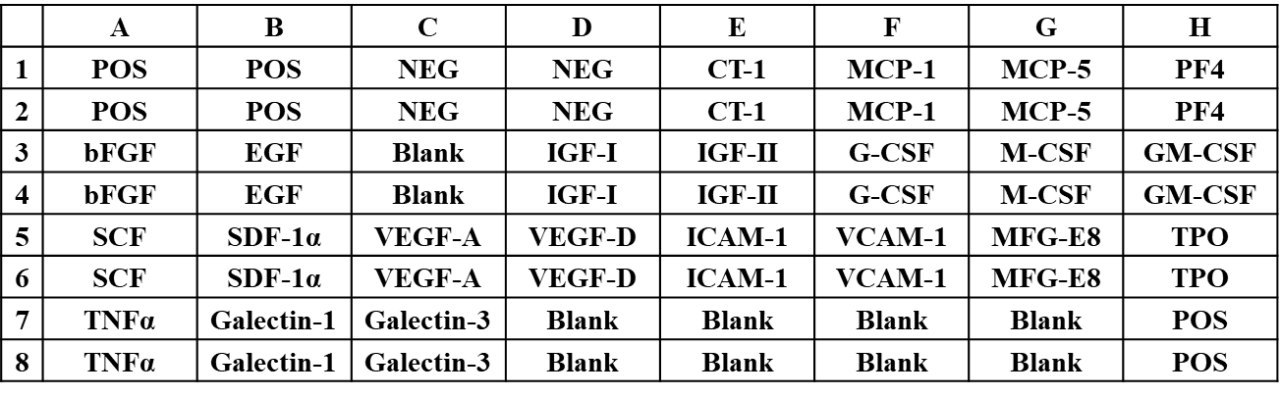


**Supplementary Figure S2.** Mouse cytokines/chemokines antibody array panels of A-GECS and S-GECS lysates.
